# Cost-Effectiveness of Opportunistic Screening and Minimal Contact Psychotherapy to Prevent Depression in Primary Care Patients

**DOI:** 10.1371/journal.pone.0022884

**Published:** 2011-08-10

**Authors:** Matthijs van den Berg, Filip Smit, Theo Vos, Pieter H. M. van Baal

**Affiliations:** 1 Centre for Public Health Forecasting, National Institute of Public Health and the Environment, Bilthoven, The Netherlands; 2 Centre of Prevention and Early Intervention, Trimbos Institute (Netherlands Institute of Mental Health and Addiction), Utrecht, The Netherlands; 3 Department of Epidemiology and Biostatistics, EMGO Institute, VU University Medical Centre, Amsterdam, The Netherlands; 4 Centre for Burden of Disease and Cost-Effectiveness, School of Population Health, University of Queensland, Herston, Australia; 5 Institute of Health Policy & Management, Institute for Medical Technology Assessment, Erasmus University Rotterdam, Rotterdam, The Netherlands; 6 Expertise Centre for Methodology and Information Services, National Institute for Public Health and the Environment, Bilthoven, The Netherlands; Chiba University Center for Forensic Mental Health, Japan

## Abstract

**Background:**

Depression causes a large burden of disease worldwide. Effective prevention has the potential to reduce that burden considerably. This study aimed to investigate the cost-effectiveness of minimal contact psychotherapy, based on Lewinsohn's ‘Coping with depression’ course, targeted at opportunistically screened individuals with sub-threshold depression.

**Methods and Results:**

Using a Markov model, future health effects and costs of an intervention scenario and a current practice scenario were estimated. The time horizon was five years. Incremental cost-effectiveness ratios were expressed in euro per Disability Adjusted Life Year (DALY) averted. Probabilistic sensitivity analysis was employed to study the effect of uncertainty in the model parameters. From the health care perspective the incremental cost-effectiveness ratio was € 1,400 per DALY, and from the societal perspective the intervention was cost-saving. Although the estimated incremental costs and effects were surrounded with large uncertainty, given a willingness to pay of € 20,000 per DALY, the probability that the intervention is cost-effective was around 80%.

**Conclusion:**

This modelling study showed that opportunistic screening in primary care for sub-threshold depression in combination with minimal contact psychotherapy may be cost-effective in the prevention of major depression.

## Introduction

Depression is a leading cause of burden of disease and health care costs [1]–[3]. Worldwide, depression ranks third on the list of leading causes of burden of disease, causing over 4 percent of all disability adjusted life years (DALYs), and it is projected to rank first on this list by 2030 [1], [2]. For middle- and high-income countries, depression was already the leading cause of burden of disease in 2004, causing over five and eight percent of all DALYs, respectively [2]. In the Netherlands, almost four percent of the burden of disease is caused by depression [4], and the 12 month prevalence of depression is 5.4% [5]. These rates compare to other European countries [6]. For the Netherlands, it is estimated that the direct medical costs of depression are 773 million euros (1.1% of total costs of illness in the Netherlands) [7].

Effective prevention of major depression has the potential to reduce the burden of disease considerably. Three types of prevention can be discerned, depending on the target group of the intervention: universal (targeted at entire populations), selective (targeted at high-risk groups), or indicated (targeted at individuals with depressive symptoms not meeting all criteria for a depressive disorder). An important reason for targeting prevention at people with sub-threshold (minor) depression is that they have an increased risk of developing major depression compared to persons not meeting the criteria of sub-threshold depression [8]. In addition, sub-threshold depression is associated with impaired functioning, reduced quality of life, and excess medical and non-medical costs [9], [10].

Various types of psychotherapy have been evaluated not only to cure depressive episodes but also to prevent first and further episodes [11]–[13]. A recent meta-analysis attributed a statistically significant reduction of 22% in the incidence of depressive disorders to psychological interventions [11]. This meta-analysis included all types of prevention (universal, selective, and indicated). A meta-analysis of indicated prevention of major depression in individuals with sub-threshold depression only, found a risk reduction of 30%, but this was not statistically significant [12]. Cognitive behavioural therapy in the form of a ‘Coping with depression’ course was in several studies found to result in a reduced risk of getting major depression of 38% [13]. So, effective prevention of major depression has the potential to reduce the burden of disease considerably.

In a Dutch trial, minimal contact psychotherapy, based on Lewinsohn's ‘Coping with depression’ course, prevented one third of the incidence of major depression in individuals with sub-threshold depression [14]. As this specific delivery format of the ‘Coping with Depression’ course requires little effort and therapists' time, it seems attractive from an economic point of view. Moreover, the bibliotherapeutical format introduces some additional benefits: it is a low threshold intervention, with no fear of stigma involved; it focuses on empowering the participants by improving self-management skills; and it can be conducted at times that agree best with the participant's agenda.

Although the evidence base for effectiveness of depression prevention is growing, evidence for its cost-effectiveness is still scarce. To our knowledge, few economic evaluations of preventive interventions for depression have been published. Smit et al. evaluated the cost-effectiveness of MCP in costs per avoided major depression episode, with a one year time horizon, not including the costs of screening for eligible participants [15]. Lynch et al. performed an economic evaluation as part of a trial in which the effectiveness of a CBT course for the prevention of depression in adolescents with depressed parents [16]. Vos et al. published cost-effectiveness analyses of several depression interventions, including relapse prevention by maintenance treatment [17]. In this paper, we estimated the costs and benefits of opportunistic screening in general practice and treatment with minimal contact psychotherapy for individuals with sub-threshold depression to prevent the incidence of depressive disorders in those individuals.

## Materials and Methods

Epidemiological modelling combines available evidence from different sources and enables predictions of future costs and benefits. Using a Markov model, we estimated the costs and benefits of an intervention scenario and a reference scenario over a five year period. The effectiveness of the intervention was modelled through a decreased transition probability from sub-threshold depression to major depression. Disability weight for the different health states enabled us to calculate the benefits in terms of DALYs averted. The model combines medical and societal costs of both minor and major depression, so the cost-effectiveness (in euros per DALY) of opportunistic screening in general practice for sub-threshold depressed individuals, and minimal contact psychotherapy to prevent depressive disorders in those individuals, was evaluated both from a health care perspective and from a societal perspective.

### Intervention

The modelled intervention is based on a single trial [14], and consists of two steps:

1. **Opportunistic screening.** Persons eligible for the intervention, i.e. persons with sub-threshold depression, were opportunistically recruited from general practice. First, people in the waiting room for a GP visit unrelated to depressive symptoms were approached by the practice assistant. Those who were eligible for screening and gave informed consent were then screened for sub-threshold depression (participation rate: 72.5%; screen positive rate: 26.6%). In a second step, screen-positive patients were approached for follow-up diagnostics in a clinical interview to exclude those who met criteria for a depression or anxiety disorder (participation rate: 35.7%; exclusion rate: 40.5%) [14].
2. **Minimal contact psychotherapy.** The intervention was based on Lewinsohn's ‘Coping with Depression’ course [13], and consisted of a self-help manual with instructions on cognitive-behavioural self-help in mood management. The manual also contains homework assignments aimed at cognitive restructuring, activity scheduling to increase pleasant activities and relaxation. Before starting reading the manual, a brief face-to-face interview with a prevention specialist or a clinician from a community mental health centre took place. Thereafter, six short telephone calls (maximum 15 min each) were offered supporting the participants in working through the manual. The effect of MCP was a one third decrease of the incidence rate after 12 months (incidence rate ratio 0.66, 95% CI 0.40–1.09, significant in the one-sided test) [14].

### Scenarios

To estimate the cost-effectiveness of minimal contact psychotherapy as indicated prevention of depression, two scenarios were compared:

- Intervention scenario: all persons in the target population are screened and those with sub-threshold depression receive minimal contact psychotherapy.
- Current practice scenario: persons in the target population are not screened, do not receive minimal contact psychotherapy, and receive care as usual from their GP.

### Target population

In this modelling study, the target population consists of all people between age 20 and 65 visiting the GP within one year. In 2008, the total Dutch population aged 20–65 years accounted for about 10 million people. Seventy-two per cent of them (i.e. 7.2 million) visited the GP at least once in that particular year (Statline database, Statistics Netherlands).

### Depression Markov Model

**Figure 1:**
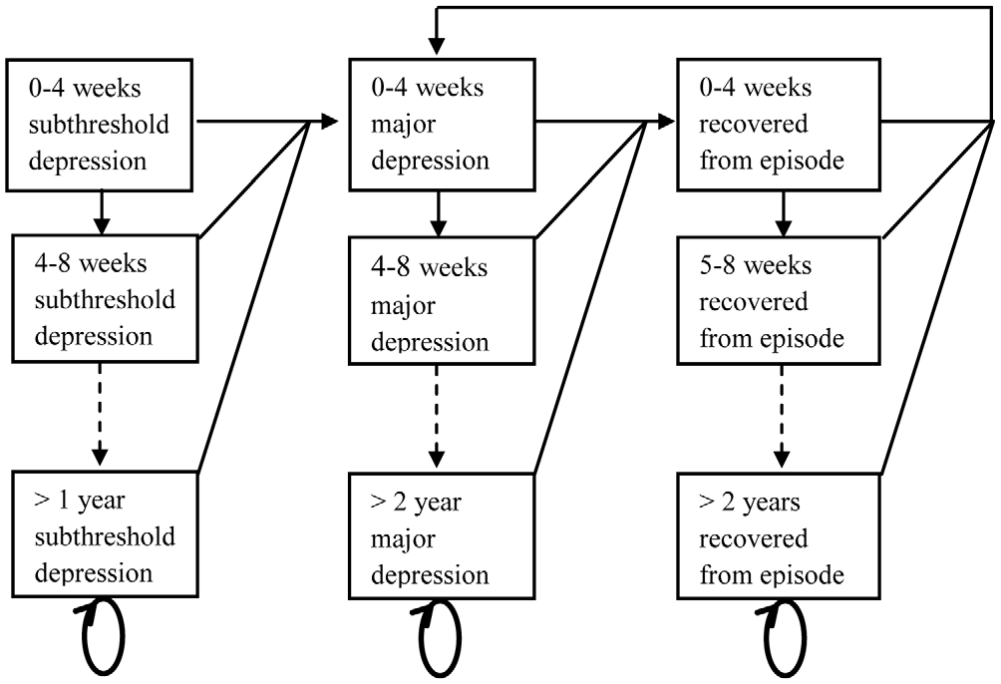
Graphical representation of the depression Markov model. The model simulates a cohort of people diagnosed with sub-threshold depression over time in cycles of four weeks.

**Figure 2:**
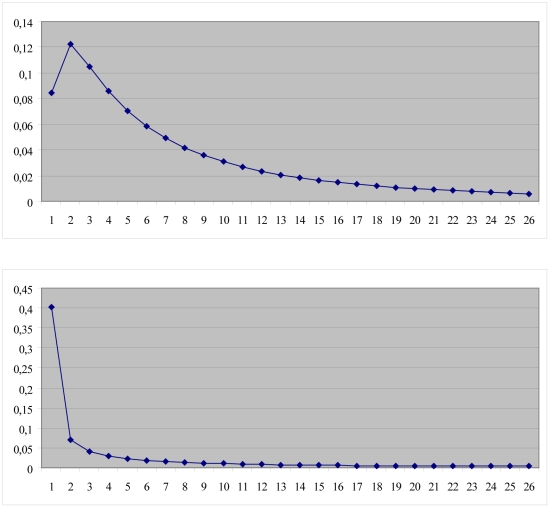
Probability curves of recovery from depression (upper graph) and relapse after depression (lower graph).

To estimate future health effects and costs a depression Markov model was developed (Figure 1). The model allows simulating a cohort of people diagnosed with sub-threshold depression over time in cycles of four weeks (0–4 weeks, 4–8 weeks, etc.). In every cycle, a person with sub-threshold depression has a probability to develop an episode of major depression, or to remain sub-threshold depressed. We assumed no remission from sub-threshold depression in the intervention and current practice scenarios. One year-probability for developing an episode of major depression was derived from the observed number of events in the control arm of the trial [14], [18]. The one-year probability was transformed into 4-week probability assuming a constant hazard rate, resulting in a probability of developing a major depressive episode of 1.6%. Accordingly, sub-threshold depressed individuals undergoing MCP (intervention scenario) have a probability to develop a major depressive episode of 1.1% each cycle of four weeks. It was assumed that the probability for persons with sub-threshold depression to develop a major depressive episode does not depend on the time spent in state but only whether they receive the intervention or not. The effects of the MCP intervention on major depression incidence and health care utilization are assumed to last only for one year. After one year, persons with sub-threshold depression use the same amount of health services and have the same probability to develop major depression in both scenarios. Once a person is in a major depressive episode, this person has a probability to either recover or to remain depressed in subsequent cycles. The probability to recover declines as the length of the episode increases. Once recovered, people have a probability to relapse into a major depression, or to remain recovered. There is a difference between relapse and recurrence. Recurrence can only occur after a person's recovery was sustained over 6 months. By contrast, relapse can occur during remission but before recovery. We collapsed both health states into a single category, using the term relapse. The probability to relapse decreases over time. Recovery and relapse curves were estimated based on data from the Dutch NEMESIS study, and an Australian modelling study [19], [20]. These probability curves are presented in figure 2. Since the follow up in the trial was one year, and the time span of both the recovery curve and the relapse curve was two years, a time horizon of five years was chosen.

### DALY's

Disability weights were derived from the Dutch disability weights study [21]. Based on this study, the mean disability weight for major depression was 0.46. Since we did not found a disability weight for sub-threshold depression in the literature, we used data from an unpublished study among a small group of Dutch physicians. Using a calibrated visual analogue scale the mean disability weight was estimated at 0.097 (95% CI 0.044–0.151) (Smit et al., unpublished data). Persons recovered from a major depressive episode were assumed to have the same level of disability weight as those with sub-threshold depression. DALYs were calculated by multiplying the time spend in each health state by the disability weight of that state.

### Costs

#### Intervention costs

As mentioned above, the screening had two steps. The first screening step, i.e. the actual screening for sub-threshold depression, was calculated to cost about €5,- per capita. The second screening step, i.e. the interview with screen-positive patients to ascertain their diagnostic status and eligibility for the intervention, costed about €119 per capita. The intervention, consisting of an intake session, a manual and the phone calls, costed €423 per capita. All intervention costs were derived from the economic evaluation of minimal contact psychotherapy as indicated prevention in primary care [15].

#### Health care and societal costs

Both health care costs and societal costs related to sub-threshold depression were based on Smit et al. Societal costs include costs of informal care, and productivity losses due to absenteeism [15]. Both health care costs and societal costs of major depression were based on three Dutch trials of different therapies for depression in which the costs associated with major depression were measured for at least one year [22]–[24]. Societal costs include costs of informal care [23], [24], productivity losses [22]–[24], patient costs (e.g. travel time) [24]. In all studies, productivity costs formed the major part of the societal costs. The mean health care and societal costs of major depression were estimated using a random effects meta-analysis of these three trials. Costs of those recovered from depression were assumed to be equal to the costs of sub-threshold depression [25]. Mean four week health care costs and societal costs for different health states are presented in table 1. All costs were indexed to 2008.

**Table 1:** Distributions of the model parameters used in the probabilistic sensitivity analysis and point estimates with confidence intervals between brackets.

		Reference scenario	Intervention scenario
***Screening process***	*Fraction of the target population that agrees to be screened*		Beta distribution^a^ (alfa = 3826; beta = 1452) 0.725 (0.713–0737)
	*Fraction of screened included for diagnostic interview*		Beta distribution^a^ (alfa = 364; beta = 3463) 0.095 (0.086–0.105)
	*Fraction of interviewed included in intervention*		Beta distribution^a^ (alfa = 217; beta = 148) 0.595 (0.544–0.645)
***Sub threshold states***	*Incidence probability from sub-threshold to major depression*	Beta distribution^b^ (alfa = 21; beta = 90) 0.016 (0.010–0.023)	Beta distribution^b^ (alfa = 14; beta = 95) 0.011 (0.06–0.017)
	*Health care costs for sub-threshold depression*	Gamma distribution^c^ (alfa = 15; beta = 108) 132 (73–207)	Gamma distribution^c^ (alfa = 31; beta = 55) 139 (94–192)
	*Total costs for sub-threshold depression*	Gamma distribution^c^ (alfa = 33; beta = 258) 439 (219–691)	Gamma distribution^c^ (alfa = 16; beta = 433) 384 (138–692)
	*Quality of life for sub-threshold depression*	Beta distribution^d^ (alfa = 106; beta = 11) 0.906 (0.847–0.952)	Same as No MCP
***Major depression states***	*Health care costs for major depression*	Gamma distribution^e^ (alfa = 13; beta = 11) 268 (150–419)	Same as No MCP
	*Total costs for major depression*	Gamma distribution^e^(alfa = 14; beta = 26) 615 (308–1022)	Same as No MCP
***Recovered from major depression states***	*Health care costs in those recovered from major depression*	Same as health care costs in sub-threshold depression states	Same as No MCP
	*Total costs in those recovered from major depression*	Same as health care costs in sub-threshold depression states	Same as No MCP
	*Quality of life in those recovered from major depression*	Same as QoL in sub-threshold depression states	Same as No MCP

a Derived from observed number of events [14], [18].

b One year-probabilities were derived from observed number of events [14], [18]. One-year probabilities were transformed into 4-week probabilities assuming a constant hazard rate.

c Yearly costs derived from Smit et al. using method of moments [15]. Yearly costs were divided by 13 and multiplied by price indices to obtain costs per cycle. Societal costs include health care costs but exclude the costs of work cut-back as reported in Smit et al. [15] as these were not included in the studies used to estimate societal costs associated with major depression [20]–[22].

d Distribution was derived from a sample of Dutch physicians' estimation of the utility of sub-threshold depression (0.903, 95%CI 0.849–0.956) (Smit et al., unpublished study).

e Yearly costs derived using random effect meta analyses from 3 studies [20]–[22]. Yearly costs were divided by 13 and multiplied by price indices to obtain costs per cycle. Societal costs include health care costs.

### Cost-effectiveness ratio

Incremental cost effectiveness ratios (ICER) are expressed in euros per DALY averted. In accordance with the Dutch guidelines, costs were discounted at 4% and effects at 1.5% [26]. The time horizon was five years. With probabilistic sensitivity analysis (5000 runs), uncertainty in the input parameters was addressed and reflected in the model output (estimated incremental costs and DALYs). The distributions used in the probabilistic sensitivity analysis are shown in table 1. The ICER was calculated as a ratio of mean incremental costs to mean incremental effects [27].

## Results

If trial results [14] were achieved in the whole population, 1.4 million persons would be screened positive for sub-threshold depression, and 0.3 million persons would receive minimal contact psychotherapy (4% of the target population). Table 2 shows the incremental costs and effects at population level. The mean incremental health care costs were estimated at €16M (95% CI: −262M/283M), and the mean incremental total costs (including societal costs) were a saving of €390M (95% CI: −1480M/813M). The intervention was estimated to avert 12,000 DALYs (95% CI: −9,000/30,000). From the health care perspective, the ICER was € 1,400 per DALY. From the societal perspective the intervention was estimated to save costs per averted DALY.

**Table 2:** Estimates of total incremental costs and effects in the target population^a^.

Incremental health care costs (€ Millions )^c^	16 ( −262/283)
Screening costs	54 (50/59)
Costs of the intervention	42 (36/47)
Other health care expenditures	−80 (−360/186)
Incremental societal costs (€ Millions)^c^	− 390 (−1480/813)
Incremental DALYs averted (thousands)^b^	12 (−9/30)
Incremental cost-effectiveness ratio, health care perspective (€ per DALY)	1,400
Incremental cost-effectiveness ratio, societal perspective (€ per DALY)	Cost saving

a Intervention scenario compared to reference scenario (95% confidence intervals between brackets).

b Discounted with 1.5%.

c Discounted with 4%.

**Figure 3:**
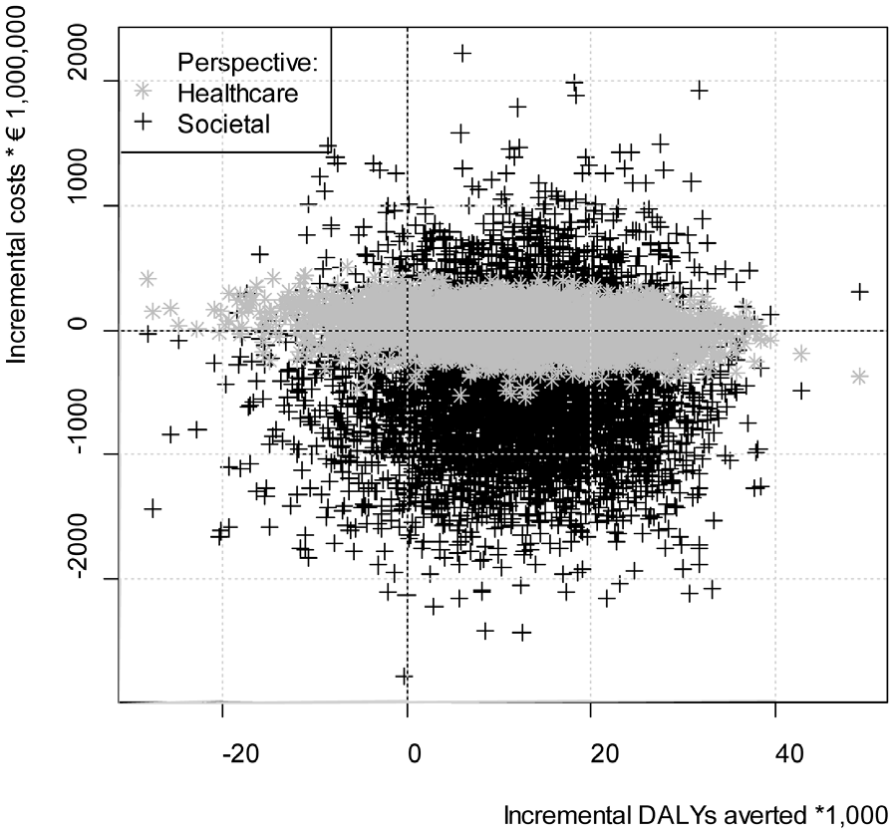
Incremental effects and incremental costs of the intervention scenario vs. the reference scenario from the health care perspective and the societal perspective.

Figure 3 displays cumulative differences in costs and effects (both discounted) of the intervention scenario compared to the current practice scenario for different values of the input parameters over a period of five years. Figure 3 shows that in both the health care perspective and the societal perspective, screening and treating sub-threshold depression can result in cost savings, additional costs, health gains and health losses. The north-east quadrant represents health gains at additional costs, the south-east quadrant represents health gains and cost savings, the south-west quadrant represents health losses and cost saving, and the north-west quadrant represents health losses at additional costs. For the health care perspective, the percentages of the points in these four quadrants were: 47% in the NE, 42% in the SE, 3% in the SW, and 8% in the NW, respectively. For the societal perspective these percentages were: 21%, 67%, 9%, and 2%.

**Figure 4:**
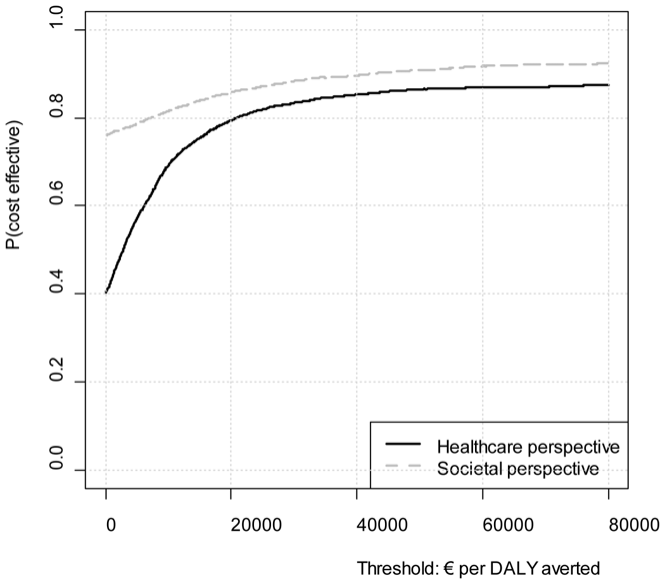
Cost-effectiveness acceptability curves for the health care perspective and the societal perspective.

Figure 4 displays the cost-effectiveness acceptability curves (CEAC). A CEAC displays the probability that an intervention is cost-effective for a range of willingness to pay thresholds. The probability that screening followed by minimal contact psychotherapy, compared to care as usual, is cost-effective increases as the threshold increases. What can be derived from Figure 4, is that if we would take the threshold of € 20,000 per DALY, as often used in the Netherlands [28], the intervention would have a probability of 79% or 83% to be cost-effective in the health care perspective or the societal perspective, respectively.

## Discussion

This modelling study showed that from a health care perspective health gains may be achieved cost-effectively if a screen-and-treat strategy for sub-threshold depression would be implemented in primary care. From a societal perspective, the modelled strategy of depression prevention was estimated to result in cost savings. There were however large uncertainties around the mean incremental costs and mean incremental effects, and the probabilistic sensitivity analysis resulted in estimates in all four quadrants of the cost-effectiveness plane.

### Strengths

Although the evidence base for effectiveness of depression prevention is growing, evidence for its cost-effectiveness is still scarce. To our knowledge, few economic evaluations of preventive interventions for depression have been published [15]–[17]. Smit et al. evaluated the cost-effectiveness of minimal contact psychotherapy in costs per avoided major depression episode, with a one year time horizon, not including the costs of screening for eligible participants [15]. In our model, the outcome measure was expressed in DALYs, a five year time horizon was used, and all relevant costs were included. Moreover, the economic evaluation was performed using both the health care perspective and the societal perspective. Although the intervention was cost-effective in both perspectives, large differences were found. From the health care perspective money needs to be invested to realise health gain, while from the societal perspective it was estimated that the intervention generates both health gain and costs savings. Substantial costs associated with depression (e.g. productivity losses) are not taken into account using a health care perspective. This underlines the necessity of incorporating all relevant costs and effects in economic evaluations. Finally, this study modelled minimal contact psychotherapy as the preventive intervention of choice, while other delivery formats of Lewinsohn's Coping (e.g. online intervention, and face-to-face intervention) are also available in the Netherlands. We believe that it is important to have different delivery formats available, such that the intervention can be offered in a format that matches the preferences and capacities of the individual patient in the best possible way.

### Limitations

As in any modelling study, we made some simplifying assumptions that deserve further research. Most importantly, we assumed that the effectiveness of the intervention would last for only one year. Furthermore, persons with sub-threshold depression who do not develop a major depressive episode within a year would remain at risk for major depression. So, we assumed no remission from sub-threshold depression. The disability weight for sub-threshold depression was based on a small, unpublished study. Higher disability weights for sub-threshold depression would result in slightly smaller health gains in the intervention scenario, and in a somewhat higher cost-effectiveness ratio. For instance, with a disability weight of 0.19, which equals the disability weight of mild major depression [21], the ICER stays below €2.000 per DALY averted (health care perspective). We also assumed that the disability weight for persons recovered from major depression is equal to the weight for persons with sub-threshold depression. Moreover, although having a disease history of depressive episodes affects ones future risk, we did not make adjustments for disease history in the model. And although the risk of relapse or recurrence increases with the number of previous depressive episodes [29], relapse probabilities were not modelled to depend on number of previous episodes of major depression. A constant hazard rate was used for the transformation of the one year probability of developing a major depressive episode into a four week probability. So, in the model, the probability for persons with sub-threshold depression to develop a major depressive episode did not depend on the time spent in the state but only whether they receive the intervention or not. It might however be the case that the longer one has sub-threshold depression, the more likely that major depression will supervene.

We did take into account the uncertainty around a lot of model parameters. Nevertheless, the uncertainty around some important parameters (e.g. relapse probabilities, and health care costs of those just recovered from depression) could not be assessed. Additional uncertainty may influence the estimates of the cost effectiveness ratio. Furthermore, assumptions concerning the effectiveness of minimal contact psychotherapy were based on a single trial, carried out in the Netherlands. The trial was underpowered, and the incidence risk ratio that was only significantly different from 1 in the one-sided test. Although this is indeed a rather small evidence base, meta-analyses on the effectiveness of psychotherapy in the prevention of depression show comparable effectiveness with incidence risk ratios between 0.62 and 0.78, significant in two-sided tests [11]–[13]. We incorporated the uncertainty surrounding the intervention's effectiveness in the probabilistic sensitivity analysis. This is reflected in the broad 95% confidence intervals of the estimated incremental benefits and costs. Based on the trial, we also assumed that every positive screen was followed by a diagnostic interview to exclude major depression or anxiety disorders. However, in everyday practice such a double check would be unrealistic. Depression is associated with increased mortality rates. However, since the trial from which we derived the effectiveness figures did not include mortality as an outcome measure, we did not include this parameter in the Markov model. By excluding this outcome, the results present an underestimation of the real cost-effectiveness of this intervention. Finally, we assumed that in the intervention scenario every patient visiting a general practitioner will be screened for sub-threshold depression. This assumption was a direct extrapolation from the effectiveness trial [14]. The feasibility of screening all GP patients can be questioned. Targeting selective screening to specific settings (e.g. nursing homes, hospitals, schools) or high-risk groups (e.g. people exposed to risk factors such as chronic illness, poverty, widowhood, small social networks) might offer a more pragmatic approach. It is worth noting that applying a lower proportion of people being screened would affect both the numerator and the denominator of the ICER in the same degree, leaving the conclusion concerning the cost-effectiveness unchanged; but would affect the estimate of the total health gain in the population as well as the total intervention costs.

### Conclusion

In conclusion, opportunistic screening of primary care patients and treating those with sub-threshold depression with an intervention that reduces the risk of developing a full-blown depression with one third could save costs to society.
